# Progressive Diffuse Osteonecrosis in a Patient with Secondary Hemophagocytic Lymphohistiocytosis

**DOI:** 10.1155/2015/730719

**Published:** 2015-11-29

**Authors:** Takashi Takahashi, Jeffrey Rykken

**Affiliations:** Department of Radiology, University of Minnesota Medical Center, Minneapolis, MN, USA

## Abstract

This is a case report with serial imaging showing progression of diffuse osteonecrosis in a patient after a diagnosis of secondary hemophagocytic lymphohistiocytosis (HLH). While bone marrow involvement in HLH has been long noted at histological evaluation and is itself one of the diagnosis criteria, to the best of our knowledge, there has been no previous publication addressing osseous image findings in a patient with HLH.

## 1. Introduction

Hemophagocytic lymphohistiocytosis (HLH) also known as hemophagocytic syndrome is a rare immune disorder of macrophages [1]. The secondary (acquired) form affects older children and adults and is most commonly associated with infection, underlying malignancy, and prolonged immunosuppression such as in patients with organ or stem cell transplantation.

Although HLH primary affects the pediatric population, it can be diagnosed in patients of all ages [2]. In earlier reports, the reported incidence HLH was believed to be low, 1.2 children per million per year [3]. More recently, this is believed to be an underestimate, and a tertiary care pediatric hospital is expected to see 1 case per 3000 inpatient admissions [4]. The incidence of HLH among the adult population is less studied with one study specifically targeting previously published cases across the world that had merely 2197 reported cases clearly meeting the criteria based on a PubMed search [5]. Regardless of the actual incidence, it still is a relatively rare diagnosis. Despite its rarity, HLH has been receiving increasing attention in the field of medicine with over 1600 articles published between 2006 and 2014, whereas there are only 354 articles preceding 2006 based on a PubMed search. Given the relatively high mortality rate, early diagnosis with appropriate management is crucial.

Diagnosis of HLH is based on the combination of clinical and laboratory findings [6]. Although there are several well documented nonspecific imaging findings among HLH patients, these findings are limited to the brain and abdomen/pelvis [1]. To our surprise, despite the histological finding of hemophagocytosis in bone marrow being one of one of the diagnostic criteria for this disease entity, to the best of our knowledge, there has been no previous publication addressing the osseous imaging findings or complications in a patient with HLH.

This is a case report of a patient who had been diagnosed with secondary hemophagocytic lymphohistiocytosis and received high dose corticosteroid, IVIG, and cyclosporine as treatment with serial imaging showing progressive osteonecrosis in bone marrow involving the spine, pelvis, hips, and shoulders. Osteonecrosis of the pelvic bone was confirmed by bone marrow biopsy and involvement of the other bones assumed based on the imaging appearance.

## 2. Case Presentation

32-year-old male with past medical history significant for type 1 diabetes, hypertension, hypothyroidism, and peripheral vascular disease initially presented to an outside institution emergency department with a one-day history of bloody diarrhea, 2-3 days of nausea/vomiting, jaundice, and altered mental status. Two weeks prior to the admission, the patient and his family members had an upper respiratory infection with symptoms/signs including low grade fever, cough, and congestion. These symptoms/signs had been gradually improving; however, 2-3 days prior to presentation in the outside emergency department, the patient started to have new symptoms of nausea and vomiting, fever, and jaundice with new onset of watery stool, which developed into bloody diarrhea one day prior to initial presentation. Upon admission at the outside institution, the patient was diagnosed with hemolytic anemia for which he was initiated on steroid therapy and received a single dose of IVIG treatment.

**Figure 1:**
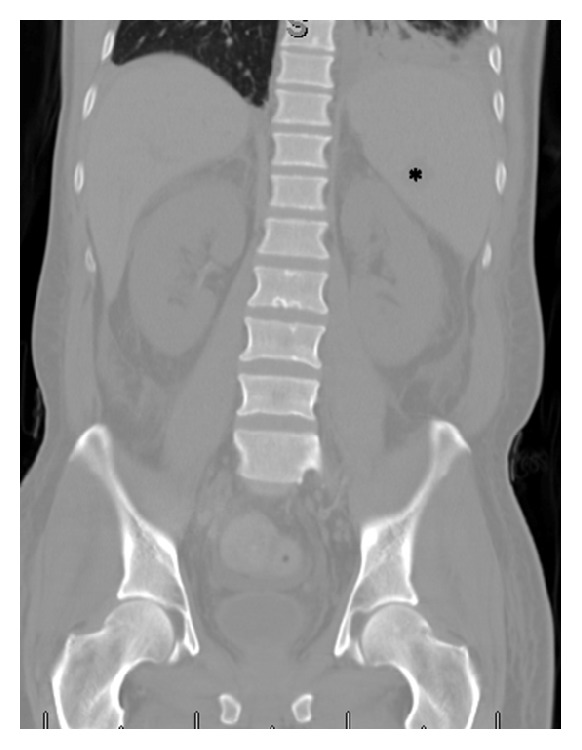
Coronal reconstruction CT in bone window at the time of admission to the outside institution shows essentially normal bone marrow other than some Schmorl's nodes. Note additional findings of splenomegaly and left basilar consolidation/atelectasis. Focus of hypoattenuation in the spleen (*∗*) was interpreted as a focus of splenic infarction. Also note that there is no evidence of osteonecrosis in either hip.

Initial CT of the abdomen and pelvis was obtained on the day of admission at the outside institution (Figure 1). At this time, CT appearance of the bone marrow was essentially normal. Following the admission, the patient rapidly developed acute renal failure, respiratory failure, altered mental status, and thrombocytopenia. Initial iliac bone biopsies/aspirations at the outside institution showed evidence of hemophagocytosis, and the possibility of HLH had been raised at this point.

For further specialized care, the patient was transferred to our tertiary institution where he had a prolonged hospitalization of over 2 months. During this hospitalization, the patient had an additional iliac bone biopsy/aspiration, which demonstrated extensive bone necrosis and again the morphologic features of hemophagocytosis. These additional biopsies were primarily performed in response to image progression of changes in the iliac bone to exclude underlying malignancy or infection, as these can be seen as a cause of secondary HLH. However, for lack of culture growth other than on an initial outside sputum culture, infectious etiologies were excluded early in this hospitalization and the patient was not placed on any antibiotics throughout the rest of the admission. No evidence of malignancy was found by laboratory, iliac biopsy, or other imaging tests. Other etiologies such as autoimmune disorder were also ruled out after a thorough clinical workup involving multiple medicine subspecialties including rheumatology, hematology, and nephrology evaluation. Laboratory evaluation included negative antiphospholipid and anti-dsDNA antibodies.

Based on clinical and laboratory criteria including bone marrow biopsy with “morphologic features of hemophagocytosis,” elevated IL-2R receptors, absent NK cell function, pancytopenia, elevated ferritin, fevers, splenomegaly, and hypertriglyceridemia, the diagnosis of HLH was clinically established. Following the HLH-2004 treatment guidelines, the patient was treated with cyclosporine and corticosteroids. Etoposide was considered per the HLH-2004 guidelines; however, it was not administered as it can cause pancytopenia in actively bleeding patients.

**Figure 2:**
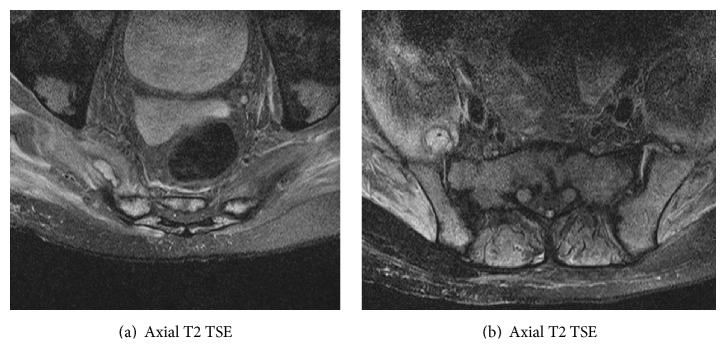
Axial T2 weighted images following a lumbosacral plexus protocol show right psoas fluid collections (a) and a fluid collection in the right iliopsoas muscle anterior to the sacroiliac joint (b). The smaller contralateral collection anterior to the left sacroiliac joint is not shown. Note diffuse muscle edema asymmetrically affecting the gluteal and iliacus musculature.

**Figure 3:**
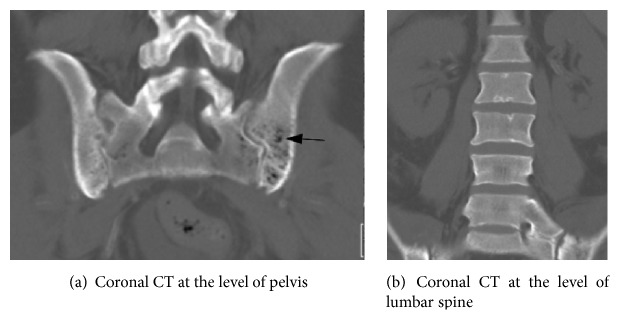
Coronal noncontrast CT three days prior to discharge/approximately 11 weeks after initial presentation shows (a) new foci of gas (arrow) within the sacrum and iliac bones, left greater than right. Findings in keeping with the preceding iliac bone marrow biopsy showing extensive bone necrosis. (b) Spine at this point still remains normal in CT appearance.

During this hospitalization, the patient had multiple noncontrast enhanced imaging studies as acute renal failure prevented the patient from receiving contrast. These imaging studies were primarily performed for evaluation of the cause of the secondary HLH and multiorgan failure. Noncontrast enhanced MRI approximately 8 weeks after the initial presentation showed fluid collections anterior to the sacroiliac joints and along the right piriformis muscle, which was interpreted as concerning for abscesses by the radiologist (Figure 2). However, based on the clinical assessment, a source of active infection seemed unlikely, and this was deemed to represent inflammatory change possibly related to adjacent bone marrow necrosis. The patient remained off antibiotics throughout this hospitalization. Three days prior to discharge (11 weeks after initial presentation), a noncontrast enhanced CT of chest, abdomen, and pelvis was obtained (Figure 3).

Approximately three months after the initial presentation (2 weeks after discharge from our institution) the patient returned to the emergency department for decreased urine output and was readmitted for acute renal failure. Although the fluid collections noted in the bilateral iliacus muscles remained relatively stable in size, given their persistence, during this second admission, the infectious disease service requested drain placement, and the patient was initiated on ertapenem therapy. The abscess culture from the right iliacus muscle fluid collection grew* Bacteroides fragilis* only in broth; nonetheless, the full course of ertapenem was completed as an outpatient.

**Figure 4:**
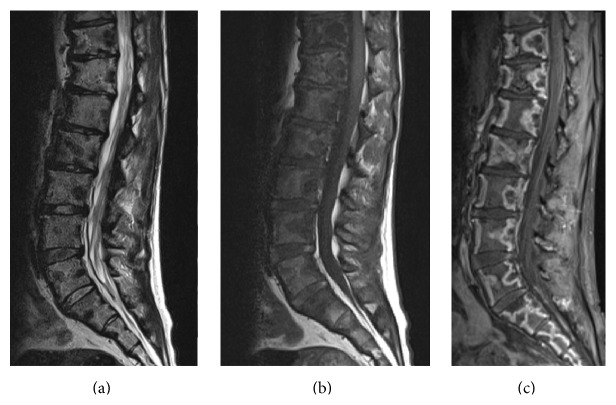
Lumbar spine MRI without and with contrast obtained 6 months after the initial presentation. (a) Sagittal T2 weighted and T1 weighted images without contrast and (c) sagittal postcontrast T1-weighted fat suppressed image. These images show marrow signal alteration with associated band-like areas of enhancement surrounding areas of nonenhancement with associated T1 and T2 hypointensity, consistent with diffuse osteonecrosis.

**Figure 5:**
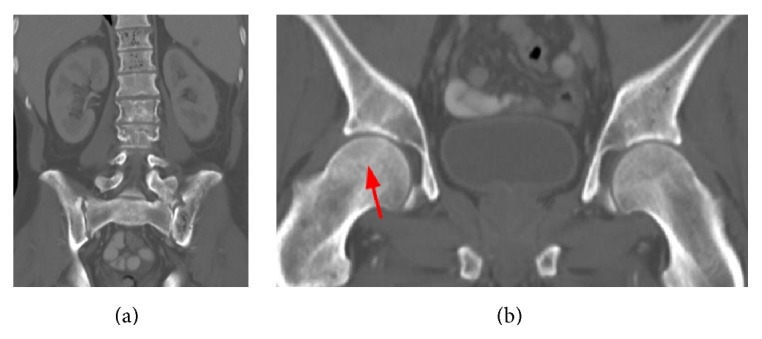
Coronal intravenous contrast enhanced CT images obtained 3 days after Figure 4. These images show (a) progression of osteonecrosis now involving the lumbar spine. (b) There is also subtle evidence of osteonecrosis of the right hip (arrow).

**Figure 6:**
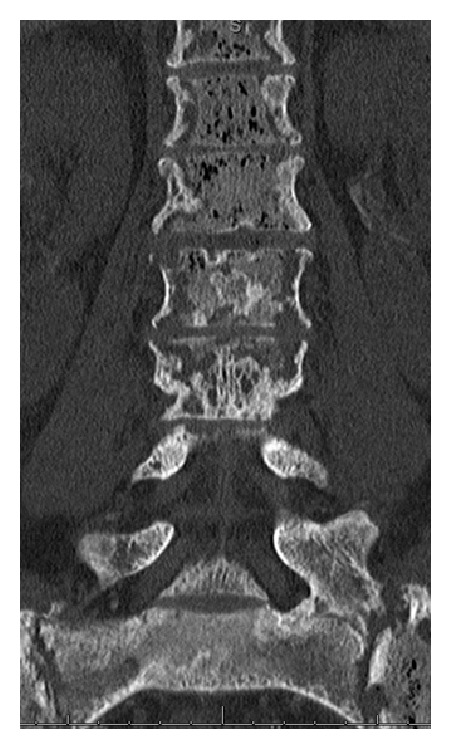
CT lumbar spine without contrast approximately 8 months since the initial presentation shows continued progression of diffuse osteonecrosis of the visualized marrow. The bone marrow is abnormal with multiple foci of gas.

**Figure 7:**
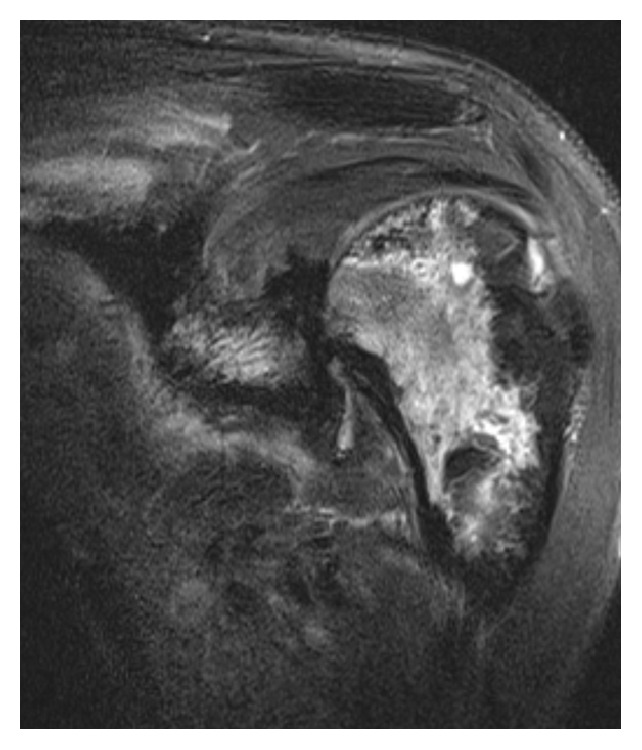
Coronal T2 weighted fat suppressed image of the left shoulder obtained approximately 1 year and 9 months since the initial presentation shows image findings consistent with avascular necrosis of the left humeral head. In this image, the classic double line sign with inner T2 hyperintensity and outer T2 hypointensity for osteonecrosis is well depicted.

**Figure 8:**
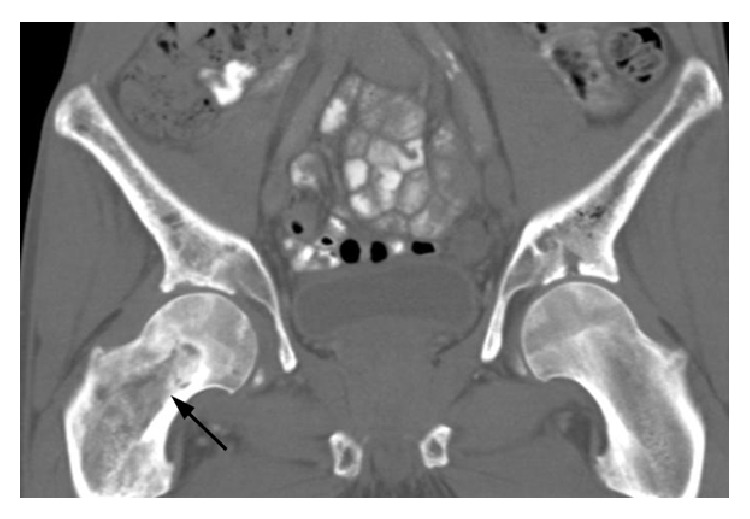
Coronal CT with oral contrast shows progression of bilateral femoral head osteonecrosis. There is also diffuse marrow signal abnormality of the visualized pelvis and right proximal femur (arrow), also believed to be consistent with osteonecrosis by imaging.

Approximately 6 months after initial presentation, the patient presented again to the emergency department with new onset of back pain and fever. MRI of the lumbar spine (Figure 4) was obtained to evaluate for discitis/osteomyelitis given the patient's prior history of possible abscess. Three days later, a CT scan of the abdomen and pelvis with contrast was also obtained (Figure 5). These images showed continued progression of osteonecrosis with worsening pelvic bone osteonecrosis and new imaging findings of diffuse lumbar spine osteonecrosis. A dedicated CT of the lumbar spine without contrast obtained approximately 8 months after the initial presentation showed further progression of the osteonecrosis (Figure 6). Due to increasing left shoulder pain, MRI of the shoulder without contrast was obtained approximately 1 year after the initial presentation (Figure 7), which demonstrated imaging evidence of avascular necrosis. Approximately 14 months after the initial presentation, the patient's abdominal CT also showed further progression of osteonecrosis involving both femoral heads (Figure 8). Furthermore, the patient also eventually developed right shoulder osteonecrosis.

## 3. Discussion

Hemophagocytic lymphohistiocytosis (HLH) also known as hemophagocytic syndrome is a rare immune disorder of macrophages, characterized by the overactivation of the mononuclear phagocytic system [1, 7]. HLH is a potentially life-threatening, hyperinflammatory syndrome caused by a lack of normal downregulation of activated macrophages and lymphocytes [8, 9].

Diagnosis of HLH requires either a molecular diagnosis or fulfilment of the combination of clinical and laboratory findings, that is, Henter's criteria. In Henter's criteria, none of the clinical or laboratory findings are specific by themselves. Therefore, five out of eight criteria must be fulfilled for the diagnosis as shown below.

*Henter's Criteria for HLH Diagnosis 2004 Version. Five* out of 8 criteria must be satisfied for the diagnosis:

 Fever.
 Splenomegaly.
 Cytopenia (affecting ≥ 2 of 3 lineages in the peripheral blood).
 Hypertriglyceridemia and/or hypofibrinogenemia.
 Hemophagocytosis in bone marrow, spleen, or lymph nodes.
 No evidence of malignancy.
 Low or absent NK cell activity.
 Ferritin level ≥ 500 *μ*g/L.
 Soluble CD 25/soluble IL-2 receptor ≥ 2400 U/mL.

HLH is divided into two forms: primary (familial) and secondary (acquired) [1, 6–8, 10]. The primary form (25% of cases) is an autosomal recessive condition associated with several genes involved in regulating the immune system, including proteins in the targeted cell death pathway. Infants and young children are most frequently affected, but the disorder is increasingly recognized in older patients [2].

The secondary (acquired) form affects older children and adults and is most commonly associated with infection, underlying malignancy, and prolonged immunosuppression, such as in patients with organ or stem cell transplantation.

HLH is a multisystemic disorder and particularly known to involve bone marrow, spleen, liver, and lymph nodes. Regardless of the form, the phenotype of the disease is the same [11]. The natural course of the disease varies depending on the cause and treatment. For example, a retrospective study showed 0% survival among patients who had EBV as a cause of HLH who had only received conservative therapy, whereas idiopathic HLH had a 100% survival rate in the same study, even with conservative therapy alone [5]. Overall the prognosis of HLH is poor in adults with a mortality rate of 41% in one study [5]. Relapse of HLH after a good therapeutic response is not uncommon; however, the actual rate and risk factors are not known. This patient has not fully recovered to his preadmission state with development of chronic renal failure, but to this date he has not had any recurrent episodes or clinical flare-ups related to HLH.

Regarding the imaging findings, several articles have been published depicting HLH associated nonspecific abnormal brain findings including diffuse cerebral and cerebellar volume loss, white matter T2 hyperintensity and calcifications, leptomeningeal and perivascular enhancement, nodular or ring enhancing parenchymal lesion, and findings of posterior reversible leukoencephalopathy [12–15]. Nonspecific pulmonary findings including atelectasis, interstitial opacities, consolidation, and pleural effusion may be seen [15]. Nonspecific abdominal findings include hepatomegaly, heterogeneous liver echogenicity, splenomegaly, marked gallbladder wall thickening, increased echogenicity of the porta hepatitis, nephromegaly, ascites, and abdominal and inguinal adenopathy [15, 16].

Despite the well documented underlying histologic involvement of bone marrow in HLH, the associated imaging changes of bone in HLH have not yet been well described [14].

In this case, we have shown progressive diffuse osteonecrosis that started in the pelvis, followed by the spine, and eventually involved the appendicular skeleton, including the hips and shoulder of a patient who had been newly diagnosed with the secondary form of HLH. Although the etiologies of the osteonecrosis are numerous, we hypothesize two potential etiologies in this clinical setting: steroid therapy and the underlying condition of HLH itself.

There is a well-documented association of steroid use and osteonecrosis, which depends on duration, daily dose, and cumulative dose of the steroid; however, the threshold is not yet known [17, 18]. As this patient had received steroids as a component of his treatment beginning on the day of admission at the outside hospital, it is not possible to totally exclude the possibility of steroids as a cause of diffuse osteonecrosis.

However, one of the bone marrow biopsies which was obtained merely 11 days after the initiation of steroid therapy had already been histologically reported as extensive osteonecrosis in the iliac bone, at which time the cumulative dose of steroid was calculated to be 6 grams of methylprednisolone. Although some studies have shown steroid induced osteonecrosis occurring as early as 36 days after the initiation of therapy, the median time between commencing steroid medication and developing osteonecrosis of the femoral head has been noted to be 18 months [18–20]. Therefore, we would consider this a rather atypically rapid manifestation and progression of osteonecrosis in this patient if steroid therapy is deemed to be the sole cause of osteonecrosis. Similarly, the extent of osteonecrosis was thought to be rather atypically diffuse for just steroid related osteonecrosis, though no literature addressing this can be found. Hence, we hypothesize the rapid progressive osteonecrosis in this newly diagnosed HLH patient is likely related at least partly to the HLH itself rather than solely from steroid use.

A limitation of this case report is osteonecrosis in the spine and appendicular skeleton diagnosed based on the classic imaging appearance without histologic confirmation other than iliac bone biopsies showing evidence of osteonecrosis. Furthermore, given that this is a single case report, the incidence of imaging findings of osteonecrosis among HLH patients is uncertain; we especially have seen a few cases with relatively normal appearing bone marrow in patients who had previously been diagnosed with HLH.
